# Genomic Characterisation of Pyometra-Associated *Escherichia coli* in a Lombardy Veterinary Clinic: A Nanopore-Based Case Series

**DOI:** 10.3390/antibiotics15020212

**Published:** 2026-02-15

**Authors:** Gabriele Meroni, Alessio Soggiu, Davide Sciannimanico, Raul Alexandru Pop, Luigi Bonizzi, Piera Anna Martino

**Affiliations:** 1 One Health Unit, Department of Biomedical Surgical and Dental Sciences, University of Milan, Via Carlo Pascal 36, 20133 Milan, Italy; gabriele.meroni@unimi.it (G.M.); luigi.bonizzi@unimi.it (L.B.); piera.martino@unimi.it (P.A.M.); 2 Freelance Veterinary Surgeon, Via A. Casati 28, 20862 Arcore, Italy; davideveter@icloud.com; 3 Department of Obstetrics and Gynecology, Faculty of Veterinary Medicine, University of Agricultural Science and Veterinary Medicine, 3-5 Mănăştur Street, 400372 Cluj-Napoca, Romania; alexandru.pop@usamvcluj.ro

**Keywords:** pyometra, dogs, cats, genomic, nanopore, virulence, antibiotic resistance

## Abstract

Background/Objectives: Pyometra is a life-threatening uterine infection of intact bitches and queens. Despite growing reports of multidrug-resistant (MDR) *Escherichia coli* in canine reproductive and urinary infections, no whole-genome data were previously available for pyometra isolates from Italy. This study aimed to characterise, by whole-genome sequencing and comparative genomics, the population structure, resistome and virulome of *E. coli* causing pyometra in companion animals from northern Italy in the context of European datasets. Methods: Four *E. coli* isolates (two canine, two feline) from pyometra cases underwent nanopore long-read sequencing. Genomes were compared with Brazilian and Finnish pyometra isolates using core- and accessory-genome analyses, pan-genome partitioning, phylogeny, and gene-based profiling of antimicrobial resistance and virulence determinants. Results: All Italian isolates belonged to phylogroup B2 and to recognised ExPEC sequence types (ST706/O51:H1, ST141/O2:H6, ST372/O75:H31, ST646/O22:H5). Phenotypically, they were uniformly resistant to several penicillins and early/third-generation cephalosporins but remained susceptible to fluoroquinolones, aminoglycosides and trimethoprim–sulphonamide. The combined 57-genome pan-genome was open yet strongly core-dominated; Italian strains shared an efflux- and regulator-centred intrinsic resistome and a rich ExPEC virulence repertoire (P, S, F1C and type 1 fimbriae, multiple siderophores, colibactin, Vat, haemolysin, CNF1) with Brazilian and Finnish isolates. Conclusions: Pyometra-associated *E. coli* from northern Italian pets belong to globally disseminated high-risk B2 lineages that combine extensive virulence with a largely intrinsic resistome, and currently retain susceptibility to several key drug classes, underscoring an important but vulnerable therapeutic window.

## 1. Introduction

In veterinary medicine, pyometra (the suppurative infection of the uterus) marks a crucial junction of microbiological complexity and clinical urgency. Mostly affecting intact female dogs and cats, *Escherichia coli* is the main pathogen found in many studies. Although polymicrobial diseases affect only 15% of animals, the microbial landscape of pyometra extends beyond a single bacterium and includes at least 32 different bacterial species in canine cases [1]. Reflecting species-specific changes in the uterine milieu and immunological responses, feline pyometra presents a distinct epidemiological picture, characterised by *Staphylococcus* spp. implicated in 8–19% of culture-positive cases [2]. Hormonal alterations during oestrus cycles (especially those driven by progesterone-mediated modifications causing cystic endometrial hyperplasia) underlie the aetiology of the disease. This structural alteration creates an environment conducive to bacterial colonisation, with ascending faecal pathogens, such as *E. coli*, exploiting cervical relaxation during diestrus [3]. Clonal persistence of uterine *E. coli* strains in concurrent bacteriuria cases, as shown by whole-genome sequencing data, points to haematogenous or lymphatic dissemination pathways [1]. With >90% sensitivity to conventional antibiotics like amoxicillin–clavulanate and enrofloxacin, antimicrobial resistance remains rare (<10% isolates) even with the prevalence of *E. coli* [4]. When compared to feline rates, which are 2.2% by 13 years, the incidence of canine pyometra reaches its highest point at 25% by 10 years [2]. There are genetic components that are evident in breed predispositions, with Sphynx and Bernese Mountain dogs exhibiting a 4.7-fold and 3.2-fold greater risk, respectively. Seasonal patterns indicate that 38% of feline cases occur in the spring, which correlates with oestrus cycles that are generated by photoperiod [5,6,7].

Over the past two decades, the epidemiology of pyometra in dogs and cats across Europe has provided significant insights into the disease’s incidence, risk factors associated with it, and breed predispositions. However, there are notable distinctions between the two species. In intact female dogs, pyometra is recognised as one of the most prevalent and deadly reproductive illnesses. It can affect as many as 25% of females that have not been spayed by the age of 10, particularly in countries like Sweden that have a high prevalence of intact animals [8]. On the other hand, pyometra in cats is less frequently recorded and researched, although it remains a significant clinical problem in intact queens [9]. In European canine populations, the incidence of pyometra is highest in middle-aged to older females, particularly between the ages of 5 and 10 years. This is due to the cumulative effect of repeated oestrous cycles and prolonged exposure to progesterone, both of which predispose the uterus to cystic endometrial hyperplasia and subsequent infection [10,11,12]. Although mixed-breed dogs make up the majority of cases due to their prevalence in clinical settings, breed predisposition has been documented. Certain large breeds, such as Rottweilers, Bernese Mountain Dogs, and German Shepherds, have been shown to have a higher risk, sometimes exceeding fifty percent in high-risk breeds [13]. Open-cervix pyometra, which typically appears with vaginal discharge, is the most prevalent form of the disease, accounting for approximately 82% of all cases [14]. Closed-cervix pyometra, on the other hand, is less common but more severe and potentially fatal [15,16]. The annual cases of pyometra vary from region to region and hospital to hospital, according to epidemiological research conducted at university veterinary clinics in Europe. These studies indicate that pyometra is responsible for a significant number of these emergency reproductive cases [9].

Additionally, environmental and reproductive factors, such as pseudopregnancy, increase the risk of developing the disease. Feline pyometra is less often diagnosed than dog pyometra, mainly because of lower clinical suspicion and different reproductive physiology. Studies, however, show that pyometra in queens mostly arises in the post-oestrus luteal phase and is caused by hormonal and bacterial elements comparable to those in dogs [17]. Seasonal trends have been noted; the spring months show a higher incidence, in line with rising cat oestrous activity [17]. Although domestic shorthair cats account for most recorded instances, indicating their demographic dominance, breed-specific data in cats are few.

Despite the clinical relevance of pyometra, comprehensive genomic studies employing long-read sequencing technologies on *E. coli* isolates from companion animals remain scarce, particularly in Southern European countries where early spaying is a common veterinary practice. In several European studies, up to 20–25% of intact bitches develop pyometra by 10 years of age, whereas ovariectomy or ovariohysterectomy virtually eliminates this risk. In Italy, as in many other European countries, elective neutering is widely recommended to prevent pyometra and other reproductive disorders, but robust, population-based incidence estimates stratified by neutering status are not yet available, which limits direct comparisons of disease frequency across countries. [6,18,19]. To date, no whole-genome sequencing studies have specifically addressed pyometra-associated *E. coli* in Italian companion animals, leaving a gap in the understanding of the local genomic landscape of these pathogens.

This study aimed to investigate the occurrence, phylogeny, virulence and antibiotic resistance of *E. coli* isolated from pyometra cases in dogs and cats from a veterinary clinic in Lombardy (Italy) in 2023–2024 using a long-reads approach.

## 2. Results

### 2.1. Antimicrobial Resistance

**Figure 1:**
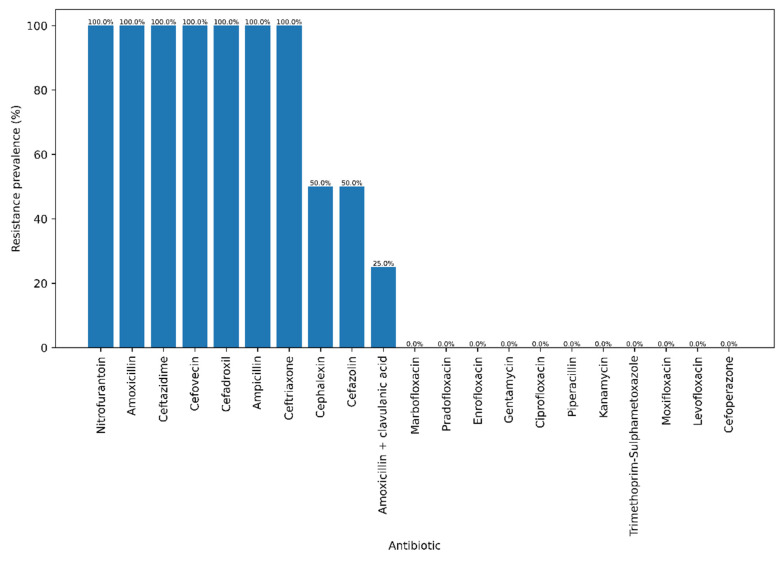
Phenotypic antimicrobial resistance profile of the four Escherichia coli isolates sequenced in this study.

Phenotypic testing (Figure 1) revealed a strikingly unbalanced resistance profile, with complete resistance to several β-lactams and no resistance to fluoroquinolones, aminoglycosides or trimethoprim–sulphonamide.

All four Italian isolates were resistant to the penicillin’s amoxicillin and ampicillin, as well as to the third-generation cephalosporin’s ceftriaxone and ceftazidime and the veterinary cephalosporin cefovecin, and showed 100% resistance to nitrofurantoin, indicating high-level non-susceptibility to first-line oral agents frequently used in companion animals. Within the β-lactam class, resistance also affected first-generation cephalosporins, with 50% of isolates resistant to cephalexin and 50% to cefazolin, whereas only one isolate (25%) remained non-susceptible to the β-lactam/β-lactamase inhibitor combination amoxicillin–clavulanic acid, suggesting that some but not all β-lactam resistance determinants can be partially overcome by clavulanate. By contrast, no resistance was detected to fluoroquinolones (enrofloxacin, marbofloxacin, pradofloxacin, ciprofloxacin, levofloxacin, moxifloxacin; 0/4, 0%), aminoglycosides (gentamicin, kanamycin; 0/4, 0%), extended-spectrum ureidopenicillin (piperacillin; 0/4, 0%), fourth-generation cephalosporin (cefoperazone; 0/4, 0%) or trimethoprim–sulfamethoxazole (0/4, 0%), indicating that the multidrug-resistance phenotype is largely confined to penicillins, early-generation and third-generation cephalosporins, and nitrofurantoin, while other major pharmacological classes remain fully active against these isolates.

### 2.2. Genetic Features of the Isolated Bacteria

The four *E. coli* strains were fully sequenced and deposited in the National Center for Biotechnology Information (NCBI) under the BioProject PRJNA1243058 (https://www.ncbi.nlm.nih.gov/bioproject/PRJNA1243058, Accessed on 15 January 2026). We have compared our results with some genomes downloaded from the literature, and more in-detail, two publications were used as a source of genomic sequences, both Finnish [1] (N = 47 genomes, PRJNA1145616 (https://www.ncbi.nlm.nih.gov/bioproject/?term=PRJNA1145616, Accessed on 15 January 2026)) and Brazilian [20] (N = 8 genomes, PRJNA891299 (https://www.ncbi.nlm.nih.gov/bioproject/PRJNA891299, Accessed on 15 January 2026)) research (Supplemental Table S1). The genomic data for the four sequenced isolates (Table 1) provide a comprehensive overview of the analysed *E. coli* strains, characterised by their origin, serotype, phylogroup, multilocus sequence typing (MLST), genome assembly level, and other genomic quality metrics.

The assembly levels range from contigs (ECOPI1) to whole-chromosomal assemblies (ECOPI2, ECOPI3, ECOPI4). ECOPI1 is characterised by a contig-level assembly with serotype O51:H1, phylogroup B2, and ST706. The genome size is approximately 4.95 Mb, with a GC content of 51%, comprising 4545 coding sequences (CDS). The assembly is non-circular, comprising four fragments, and exhibits 100% completeness with minimal contamination (0.49%). ECOPI2 is derived from a feline subject, with a chromosome-level assembly characterised by serotype O2:H6, phylogroup B2, and sequence type ST141. The genome size is higher (5.13 Mb), with a comparable GC content of 51%, and contains 4712 CDS. The genome is circular, characterised by 139× coverage, and features a high-quality assembly with perfect circularization. The completeness is 100% with contamination below 1% (0.95%). ECOPI3, derived from a dog, possesses a chromosome-level assembly, serotype O75:H31, phylogroup B2, and ST372. The genome size is approximately 5.15 Mb, with a GC content of 50%, and has 4755 CDS. The assembly is circular, exhibiting 100% completeness, and a slightly elevated contamination level of 1.37% with 135× coverage. ECOPI4, originating from a canine, exhibits serotype O22:H5, belongs to phylogroup B2, and is classified as ST646, featuring a chromosome-level assembly. The genome size is 5.25 Mb, with a GC content of 50% and 4827 CDS. This assembly is circular, with the lowest coverage (86×). The completeness is 100% with 0.85% of contamination.

### 2.3. Pan-Genome Structure and Analysis

**Figure 2:**
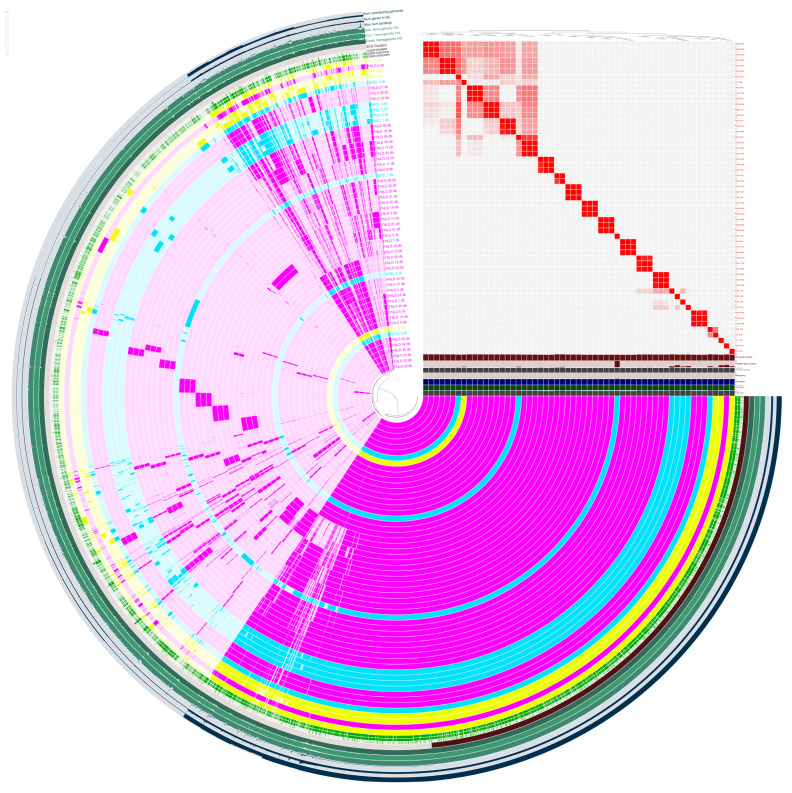
Pan-genome structure and geographic stratification of 57 *Escherichia coli* genomes. Circular pan-genome representation generated with Anvi’o showing the presence/absence of 8522 MCL-defined gene clusters across the analysed genomes. Inner rings correspond to strict core and soft-core gene clusters, while outer rings depict shell- and cloud clusters, highlighting extensive accessory genome variability. The ANI heatmap reports pairwise gene-content similarity, with off-diagonal blocks indicating groups of closely related genomes. Genomes are ordered and colour-coded by geographic origin (FNLD in lilac, BRZL in sky blue, ITLY in yellow), illustrating that accessory gene clusters tend to form region-associated patterns superimposed on a conserved core genome.

The analysis of the *E. coli* pan-genome (Figure 2) was conducted using the Anvi’o pipeline [21], providing an in-depth understanding of the genomic diversity and evolutionary dynamics of the four *E. coli* isolates compared to the Finnish (N = 47) and Brazilian (N = 8) strains. In order to compare the strains by isolation origin, Italian strains were renamed as the following: ECOPI1 = ITLY_1, ECOPI2 = ITLY_2, ECOPI3 = ITLY_3, ECOPI4 = ITLY_4.

The 57 *E. coli* genomes investigated using MCL clustering of translated coding sequences (inflation value of 10) resulted in a pan-genome consisting of 8522 unique gene clusters. The Anvi’o pan-genome summary statistics indicate that these clusters contained 12,137 gene clusters, including 3061 strict core genes (present in ≥99% of genomes) and 195 soft core genes (present in 95–99% of genomes), collectively establishing a highly conserved genomic backbone. The accessory genome contributed to the remaining diversity, including 2249 shell genes, present in 15–95% of genomes, and 6632 cloud genes, limited to less than 15% of the isolates. In the Anvi’o display, these categories are depicted as a dense, nearly continuous central signal for core and soft-core clusters, reflecting significant gene-content plasticity influenced by gain-loss dynamics and horizontal acquisition of mobile elements. The pairwise average nucleotide identity (ANI) heatmap exhibited a non-random structure. When categorised by sampling origin, these blocks aligned with geographic divisions, with FNLD, BRZL, and ITLY isolates constituting partially distinct submatrices, each defined by unique combinations of shell- and cloud clusters that were either under-represented or absent in the other regions.

**Figure 3:**
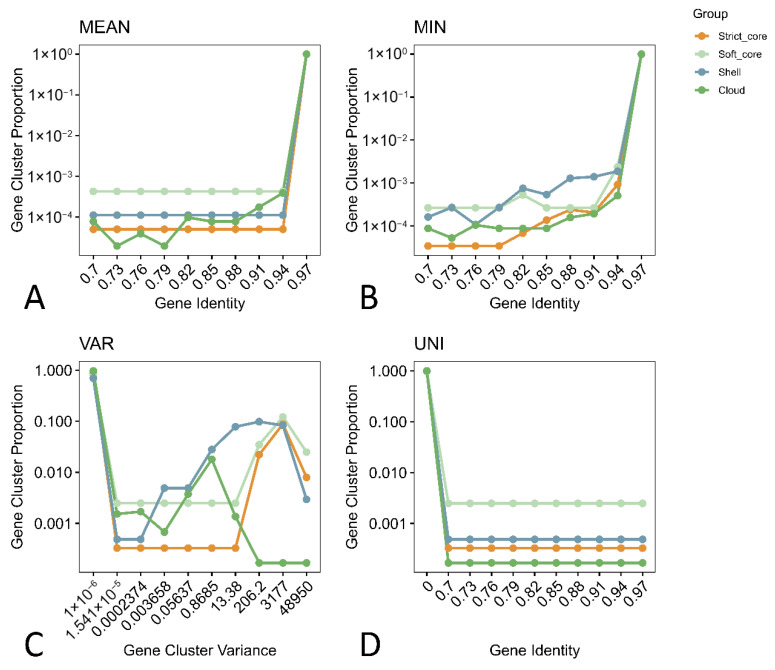
Sequence conservation and diversity of core and accessory gene clusters in the pan-genome of the analysed *E. coli* strains. Panels (**A**) “MEAN” and (**B**) “MIN” show the distribution of mean and minimum pairwise amino-acid identities across clusters, panel (**C**) “VAR” shows the variance in identity, and (**D**) “UNI” the proportion of unique clusters, stratified by pan-genome compartment. Strict-core genes exhibit uniformly high identity and low variance, whereas shell- and cloud families display broader identity ranges, higher variance and a greater fraction of unique clusters, consistent with stronger purifying selection on essential functions and extensive diversification in the accessory genome.

The statistics plot obtained with PGAP2 pipeline (Figure 3) summarises how sequence identity, variability and uniqueness differ among strict-core, soft-core, shell, and cloud gene sets. The 3A and 3B panels show that strict- and soft-core clusters maintain high mean and minimum pairwise identities, whereas shell- and cloud genes occupy a broader identity spectrum and include families with substantially lower similarity, reflecting more frequent horizontal transfer and functional diversification in the accessory genome. The variance panel (3C) reveals that sequence diversity is lowest in strict-core genes and increases progressively towards shell- and cloud clusters, which contain families with up to four orders of magnitude higher variance in identity, again consistent with strong purifying selection on essential functions and relaxed constraints on accessory loci. The 3D panel indicates that unique clusters (those represented by a single sequence) are heavily enriched in the cloud compartment and rare in the strict core, emphasising that most strain-specific innovations reside in low-frequency families.

**Figure 4:**
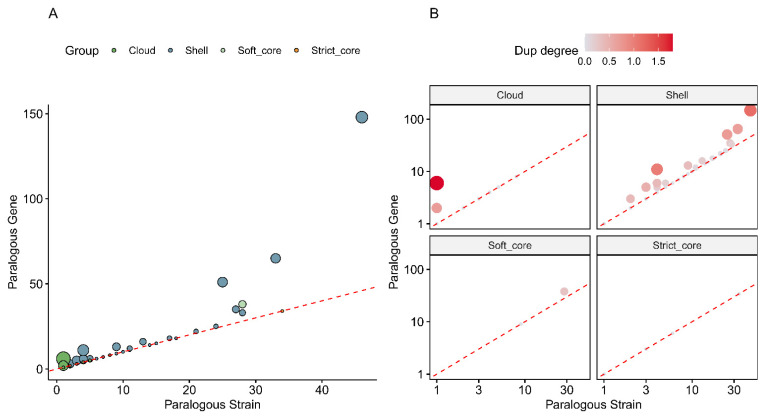
Distribution of paralogous gene copies across core and accessory components of the *E. coli* pan-genome. (**A**) Number of paralogous genes per pan-genome group as a function of the number of strains carrying paralogs, indicating that paralogs are enriched in shell- and cloud clusters. (**B**) Relationship between numbers of paralogous genes and strains, expressed as duplication degree, showing that strict-core and soft-core families are largely single-copy, whereas duplication is concentrated in the accessory genome. These results suggest that gene duplication and expansion primarily affect non-essential, accessory functions.

The paralogy statistics (Figure 4) visualise how gene duplication is distributed across the pan-genome. In panel A, the number of paralogous genes per group is plotted against the number of strains carrying paralogs, showing that paralogy is most frequent in shell- and cloud clusters, while strict-core and soft-core families harbour fewer duplicated copies and are duplicated in fewer strains. This indicates that gene amplification is predominantly an accessory-genome phenomenon, likely linked to mobile elements, metabolic flexibility and adaptive functions rather than core housekeeping roles. Panel B relates the number of paralogous genes to the number of paralogous strains via a duplication-degree measure, again demonstrating that duplication is concentrated in cloud and, to a lesser extent, shell genes, with strict-core families remaining largely single-copy.

**Figure 5:**
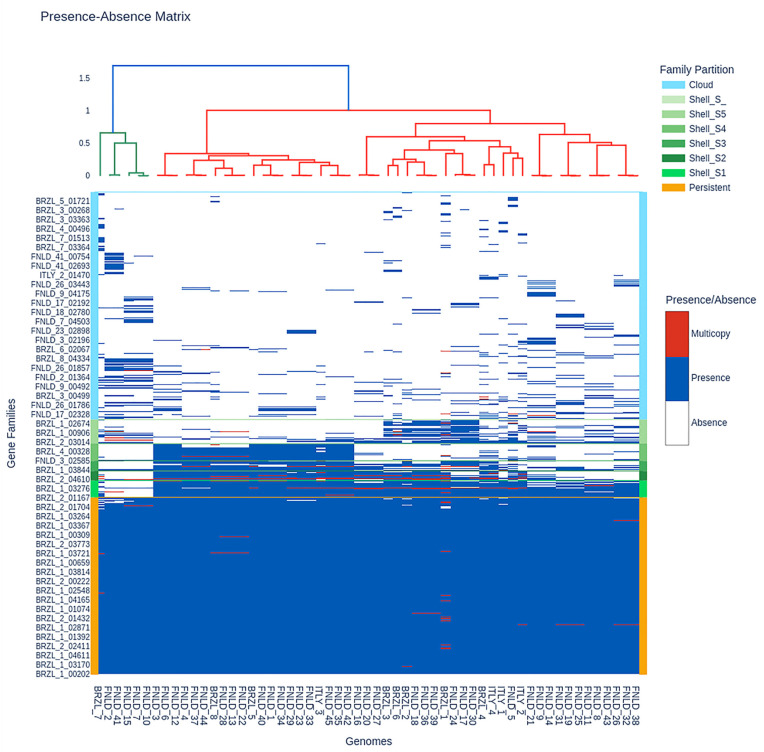
Presence–absence matrix of 10,756 gene families inferred by PPanGGOLiN across 57 *E. coli* genomes. Columns represent genomes, ordered by hierarchical clustering, and rows represent gene families coloured by partition (cloud, shell subclasses S1–S5, persistent). Blue cells denote single-copy presence, red cells multicopy presence and white cells absence. The large continuous block of persistent families contrasts with increasingly fragmented shell- and cloud regions, highlighting extensive accessory genome variability and revealing geography-associated patterns, with BRZL, FNLD and ITLY genomes forming distinct clusters enriched for specific subsets of non-persistent families.

PPanGGOLiN’s tile plot (Figure 5) displays the presence–absence matrix of 10,756 predicted gene families partitioned into persistent, shell- and cloud components [22]. The persistent partition encompasses 3061 families present in at least 55–57 genomes (orange band). Above this, approximately 3000 shell families (green bands) occur in intermediate frequencies, and appear as horizontal streaks restricted to subsets of strains, many of which correspond to the main BRZL- and FNLD-dominated clades resolved in the genome dendrogram, indicating lineage- and geography-associated accessory families. The uppermost third of the matrix is enriched in about 4500 cloud families (light blue), which are present in fewer than 10 genomes and often limited to one or a few BRZL or ITLY isolates. BRZL genomes, which constitute roughly half of the dataset, show the highest density of shell- and cloud presences per genome (frequently >1000 families), whereas FNLD genomes carry comparatively fewer rare families and cluster together with a more compact accessory profile; ITLY isolates are intermediate, sharing part of the BRZL-enriched shell repertoire but harbouring their own sets of low-frequency cloud families, suggesting distinct but partially overlapping gene pools in the three geographic groups.

**Figure 6:**
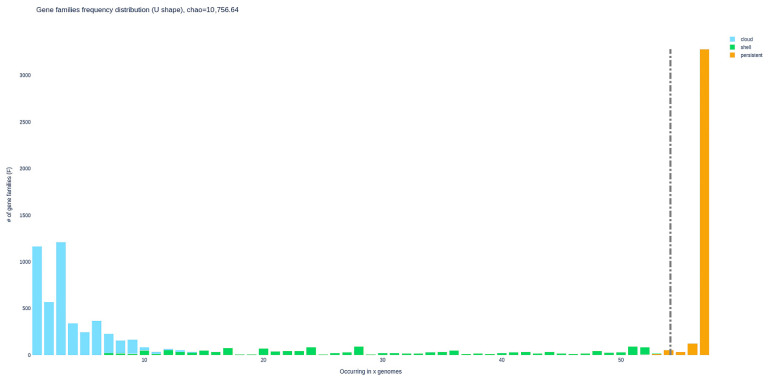
U-shaped frequency distribution of the gene families in the *E. coli* pan-genome. The x-axis reports the number of genomes in which a family occurs, and the y-axis the number of families in each frequency class. Bars are coloured by partition: cloud (light blue), shell (green) and persistent (orange). The high counts of low-frequency cloud families and high-frequency persistent families, together with a smaller shell component at intermediate frequencies, highlight an open pan-genome in which numerous rare, often geography-restricted gene families coexist with a large, highly conserved core. The vertical dashed grey line marks the presence threshold used to define the soft-core component, i.e. gene families that are present in almost all genomes.

The U-shaped frequency plot (Figure 6) shows that the *E. coli* pan-genome is dominated by rare and ubiquitous gene families, consistent with an open and strongly structured pan-genome. Among the 10,756 estimated families, more than 2200 cloud families are present in fewer than 5 genomes and about 1200 additional cloud families occur in 5–10 genomes, together representing over 30% of the pan-genome and capturing strain and region-specific innovations. The persistent partition comprises 3061 families occurring in ≥55 genomes (orange bar), accounting for ~28% of all families but >80% of the gene content of each genome, underscoring the dominance of a conserved core across BRZL, FNLD and ITLY isolates. The shell fraction, roughly 2400 families occurring in 10–55 genomes, fills the shallow middle of the U-curve and is particularly enriched in families with frequencies between 15 and 30 genomes. These distributions indicate that BRZL genomes contribute disproportionately to the low-frequency tail (owing to numerous BRZL-specific cloud families), whereas FNLD and ITLY isolates are relatively more represented in the intermediate-frequency shell classes, mirroring the patterns observed in the presence–absence matrix and sup-porting the existence of geographically structured accessory gene pools.

### 2.4. Clusters of Orthologous Groups (COG) Functional Classification

**Figure 7:**
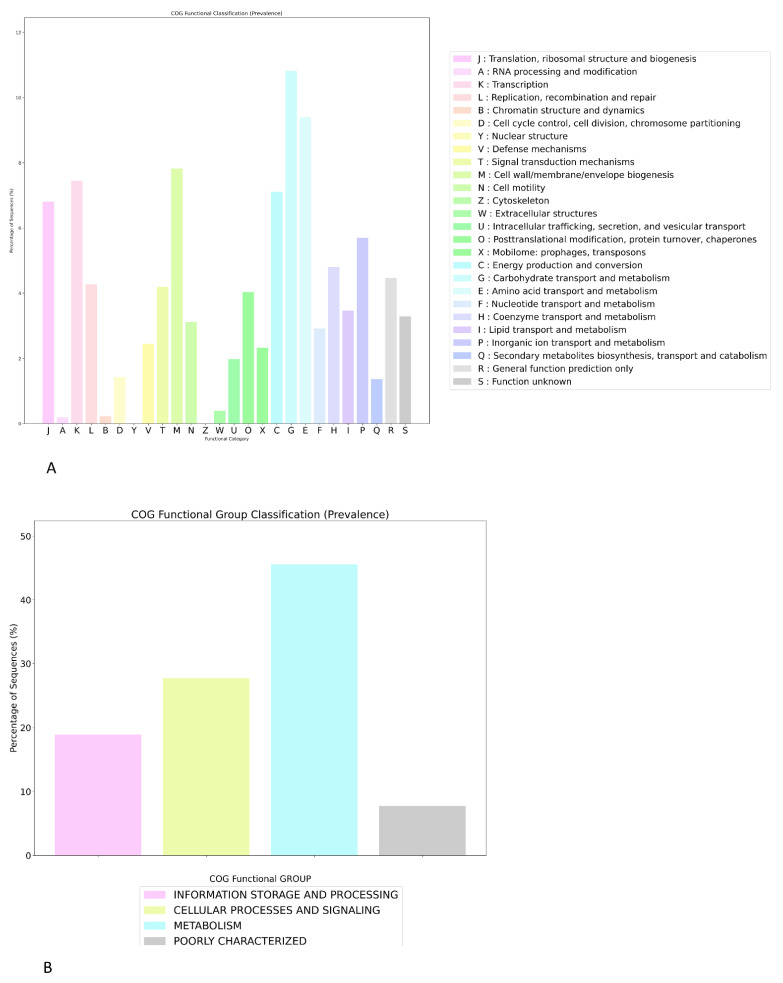
Distribution of COG functional groups (**A**) and prevalence (**B**) of individual COG functional categories across the *E. coli* pan-genome. Bars represent the percentage of sequences assigned to each of the 25 COG categories (J–S), grouped by colour into the four high-level functional groups. The plot highlights the dominance of information-processing, cell-envelope and motility functions, together with rich amino acid and carbohydrate metabolism, and shows that only a small fraction of proteins fall into poorly characterised categories.

Across the genomes, COGclassifier (Figure 7) assigned the majority of proteins to metabolic functions, which accounted for approximately 45–46% of all classified sequences, followed by cellular processes and signalling (around 27–28%) and information storage and processing (about 18–19%), whereas only ~8% of the proteins were poorly characterised. This indicates that nearly half of the coding capacity of the analysed genomes is devoted to energy production, transport and biosynthetic pathways, consistent with an expansive metabolic repertoire that likely underpins the ecological breadth observed among BRZL, FNLD and ITLY isolates.

The contribution of cellular processes and signalling suggests that more than one quarter of the proteome is dedicated to cell-envelope biogenesis, motility, stress responses and regulatory networks, which are key targets for adaptation to host- and environment-specific conditions. The lower proportion of information-storage functions implies that core replication, transcription and translation machineries are numerically minor but highly conserved, in agreement with the pan-genome analyses that identified a relatively compact persistent backbone. The limited fraction of poorly characterised proteins indicates that the functional landscape of these genomes is already well-annotated, facilitating downstream interpretation of gene-content differences between geographical groups. The distribution of 25 COG functional categories shows that translation, ribosomal structure and biogenesis (category J) is the single most represented class, encompassing roughly 6.8–7.0% of all annotated proteins, followed by transcription (K, ~7.4%) and replication, recombination and repair (L, ~4.2%). Within the cellular processes and signalling group, cell wall/membrane/envelope biogenesis (M) and signal transduction mechanisms (T) contribute ~7.8% and ~2.4% of proteins, respectively, while cell motility (N) and intracellular trafficking/secretion (U) represent a further ~4.0% and ~2.3%. Metabolic categories are also broadly represented, amino acid transport and metabolism (E) carbohydrate transport and metabolism (G) account for about 10.8% and 7.1% of proteins, respectively, with additional contributions from nucleotide (F, ~4.9%), coenzyme (H, ~3.5%), lipid (I, ~1.4%) and inorganic ion transport and metabolism (P, ~3.3). Genes involved in energy production and conversion (C) constitute ~2.9% of the proteome, whereas mobile elements such as prophages and transposons (X) contribute ~2.4%, consistent with the presence of regions of genomic plasticity that were identified as shell and cloud in the pan-genome analysis. Finally, general function prediction only (R, ~4.4%) and function un-known (S, ~3.3%).

### 2.5. Phylogenesis

**Figure 8:**
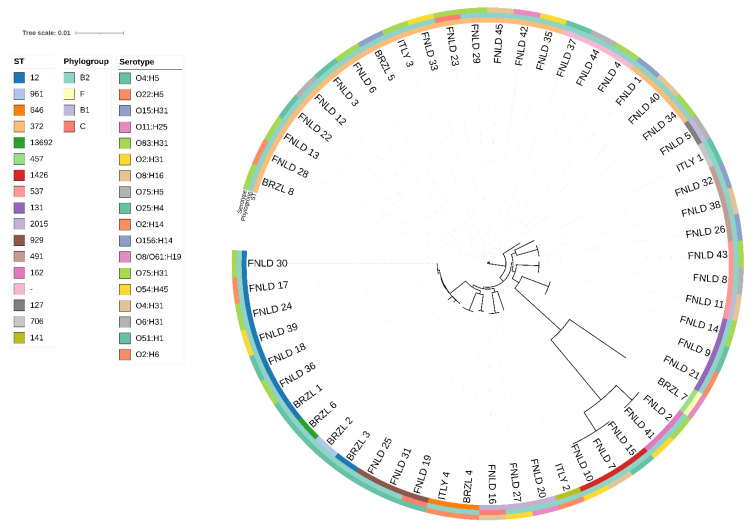
Radial maximum-likelihood phylogeny of 57 *E. coli* genomes inferred from the Panaroo core-gene alignment. Tip labels indicate individual isolates coloured by sampling origin (BRZL, FNLD, ITLY), and concentric rings report multilocus sequence type (ST), phylogroup and O:H serotype. The tree illustrates that isolates from different countries frequently cluster together within the same clades, reflecting shared clonal lineages despite geographical separation.

The radial maximum-likelihood tree (Figure 8) reconstructed from the alignment of 3061 strict core genes clusters the analysed genomes into several well-supported clades that only partially segregate by geographic origin, indicating that Brazilian, Finnish and Italian isolates belong to an interconnected global population rather than to country-restricted lineages. FNLD genomes form two major B2-dominated clusters, consistent with an overrepresentation of extra-intestinal pathogenic lineages in the Finnish set, whereas BRZL and ITLY isolates are interspersed among these clades and share identical or closely related sequence types and serotypes (e.g., O4:H5 or O25:H4) with FNLD strains, pointing to repeated international dissemination of the same clonal complexes. The concordance between phylogroup, ST and serotype rings across the tree suggests that deep branching patterns are primarily driven by vertical inheritance of the core genome, while the limited country-specific clustering implies that the marked differences observed in the accessory genome and RGP content between BRZL, FNLD and ITLY collections arise largely from recent, locally shaped gene-content flux superimposed on shared evolutionary backbones.

**Figure 9:**
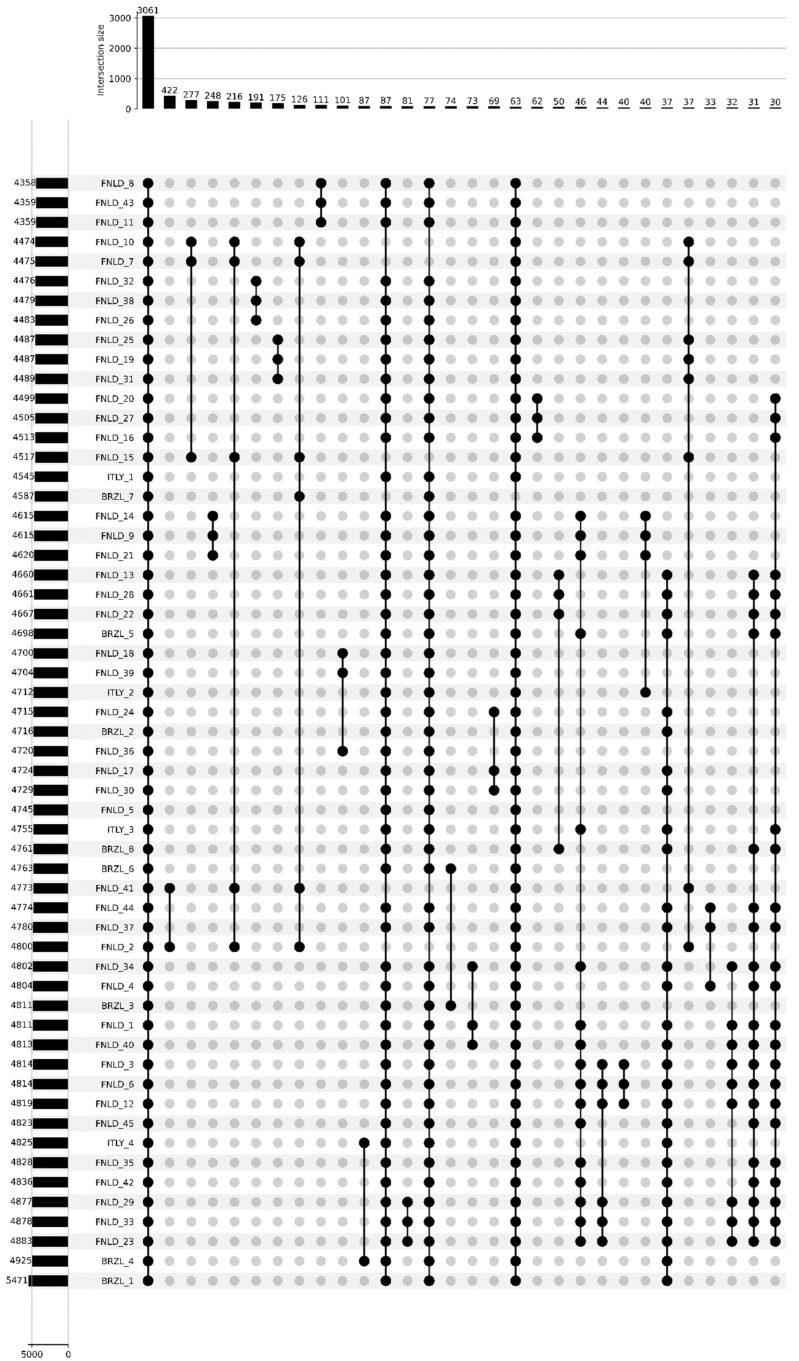
Upset plot of gene-cluster intersections for the analysed genomes. The left bar plot reports the total number of gene clusters detected in each genome, while the upper bar plot shows the size of intersections between sets of genomes indicated by connected black dots in the matrix below; the tallest bar corresponds to the 3061 clusters constituting the strict core genome. The picture was scaled to allow the representation of minimum 30 gene-cluster intersections.

The Upset diagram (Figure 9) summarises the distribution of gene-cluster intersections across the genomes, highlighting a dominant core of 3061 clusters shared by all isolates and a highly structured accessory component. The bar plot of genome-wise totals shows that each strain harbours between 4300 and 5400 clusters, with BRZL genomes generally at the upper end of this range, in agreement with their more expansive shell- and cloud fractions. Intersection bars above the matrix reveal that, beyond the universal core, the largest shared subsets comprise 422,277 and 248 clusters present in specific groups of 10–20 genomes, typically corresponding to major clades in the phylogeny and often dominated by isolates from a single country (e.g., Finnish B2 lineages or Brazilian clusters), whereas progressively smaller intersections of 40–80 clusters define more restricted combinations, frequently linking a few BRZL and ITLY genomes or singletons.

**Figure 10:**
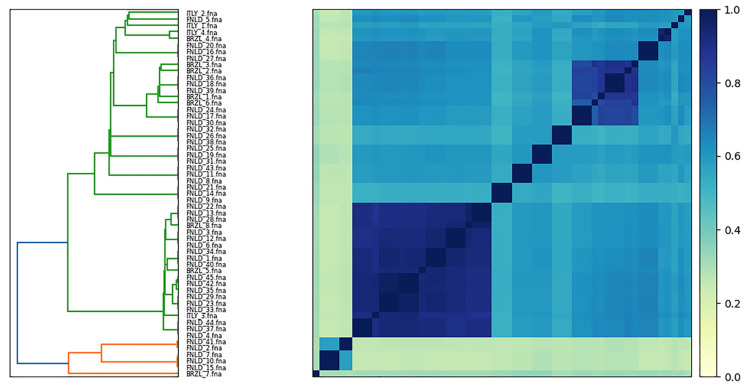
Whole-genome similarity matrix of *E. coli* computed with sourmash using sketch-based Jaccard indices. The right panel shows the similarity matrix, with darker colours indicating higher similarity and lighter colours lower similarity; the left panel displays a hierarchical clustering dendrogram based on the same distances. Two main blocks of high similarity identify closely related clonal groups, including a large cluster dominated by FNLD isolates and a smaller cluster containing BRZL and ITLY genomes, whereas a set of outlier strains forms a low-similarity band at the bottom of the matrix, reflecting pronounced whole-genome divergence.

Figure 10 summarises whole-genome relatedness among the 57 isolates using sourmash-derived clustering, revealing two major clusters of nearly identical genomes and a third, more divergent group. The ANI matrix shows a large block of pairwise identities consistently above ~99% for a subset of FNLD isolates, indicating they belong to a tightly related clonal complex, while a second, smaller high-ANI block (>99%) groups several BRZL and ITLY genomes together, consistent with the presence of internationally distributed lineages that transcend geographic boundaries. In contrast, genomes forming the pale, low-ANI band at the bottom exhibit identities dropping towards ~95–96% against the rest of the collection, marking them as outlier lineages that are still within the species boundary but substantially more distant at the core-genome level.

### 2.6. Resistome and Virulome

#### 2.6.1. Resistome

**Figure 11:**
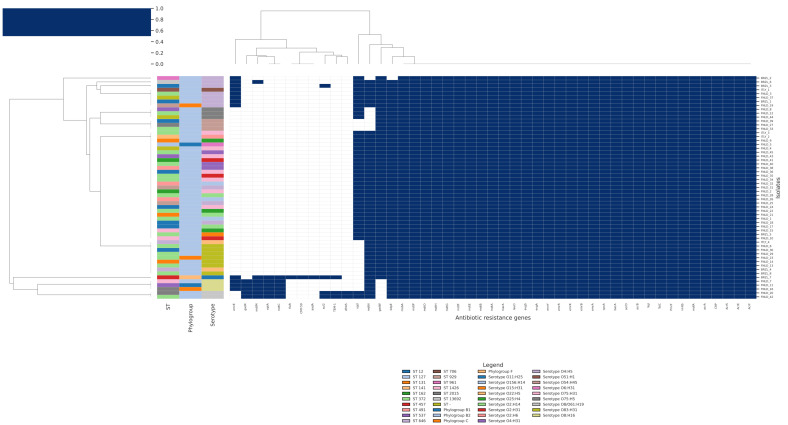
Hierarchical clustering heatmap showing the distribution of antibiotic resistance genes across all bacterial isolates, annotated by MLST sequence type, phylogroup, and serotype. Heatmap shows the presence (dark blue) and absence (white) of antibiotic resistance genes across isolates, ordered according to hierarchical clustering of the AMR profiles.

The analysis of the AMR heatmap (Figure 11) highlights a resistome that is broadly conserved across the Italian, Brazilian and Finnish genomes, with only subtle and statistically fragile signatures of geographic structuring driven by rare acquired genes. The large, continuous blue block corresponds to efflux pumps, global regulators and cell-envelope modifiers that are present in essentially all isolates, irrespective of origin. These include the AcrAB-TolC system and its accessory components (*AcrE/F/S*, *acrA/B/D*, *TolC*), multiple Mdt family pumps (*mdtA/B/E/F/G/H/N/O/P*), the MFS transporter *mdfA*, the lipid A flippase *msbA*, the bacitracin resistance factor *bacA*, and regulatory genes such as *CRP*, *H-NS*, *marA*, *leuO*, *evgA/evgS*, *cpxA*, as well as *PmrF* and *ugd*, which are involved in lipid A modification. The near-universal presence of this intrinsic backbone across all three countries indicates that baseline multidrug tolerance mechanisms are a core feature of these *E. coli* lineages and do not depend on the geographic context in which they were sampled.

Acquired resistance genes are patchily distributed, forming thin vertical bands in the heatmap that mark only a handful of isolates. *TEM-1* and *CMY-59* (β-lactamases), *sul2* and *dfrA5* (sulphonamide/trimethoprim resistance), *aadA* (aminoglycoside modifying enzyme), *floR* (phenicol exporter), and tetracycline efflux determinants *tet(A)* and *tet(B)* all occur at low frequencies, typically in 1–4 out of 57 genomes.

The chi-square analysis was used to evaluate whether any single gene shows a statistically significant association with country of origin. For the majority of loci, the chi-square statistic is low and the *p*-value is above significant threshold (e.g., *TEM-1*, *p* = 0.57; *dfrA5*, *p* = 0.76; *tet(B)*, *p* = 0.71; *gadW* and *gadX*, *p* = 0.48). This result reinforces the impression from the heatmap that the presence of most acquired determinants is compatible with random dispersal across countries, likely reflecting sporadic acquisition events followed by limited clonal expansion rather than regionally derived resistomes. A small subset of genes, however, does achieve significance when stratified by country. *CMY-59*, *aadA*, *floR*, *tet(A)* and *kdpE* all share the same chi-square statistic (χ^2^ = 6.23) and *p*-value = 0.044, while *emrE* shows a higher χ^2^ (9.92) and a more significant *p*-value = 0.007). This could indicate that particular mobile elements (e.g., plasmids carrying *CMY-59*, *aadA*, *floR* and *tet(A)*) have been introduced and propagated preferentially in one country’s population.

The sraX pipeline (Supplemental Figure S1) shows that antimicrobial resistance is dominated by a conserved scaffold of efflux- and regulator-mediated mechanisms, with modest, origin-linked variation in acquired genes and in the burden of putative resistance-associated mutations. In the first plot (Supplemental Figure S1A), which summarises the proportion of drug classes represented by unique ARGs per genome, most Brazilian, Finnish and Italian isolates display very similar ARG class profiles: chromosomally encoded multidrug efflux systems and intrinsic AmpC-like β-lactamases contribute large fractions of aminocoumarin, fluoroquinolone, macrolide, cephalosporin and peptide resistance categories in every genome, whereas classical acquired determinants (e.g., plasmidic β-lactamases, aminoglycoside, sulphonamide, trimethoprim and tetracycline genes) occur at a low frequency and expand drug-class coverage only in a subset of strains. The second plot (Supplemental Figure S1B) focuses on the subset of ARGs carrying putative SNPs linked to resistance and shows that most genomes harbour few such variants and that these are overwhelmingly located in chromosomal protein-coding genes and regulatory loci, with far fewer mutations annotated in rRNA genes or overexpression-linked determinants.

**Figure 12:**
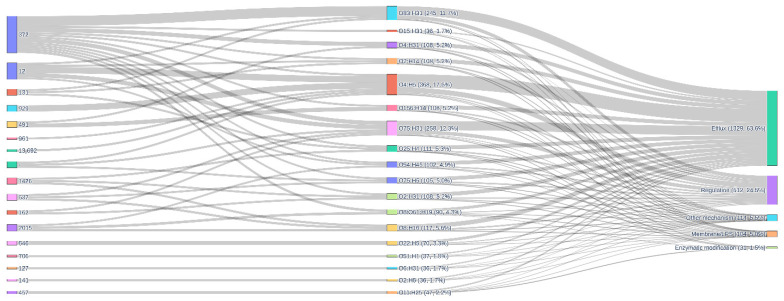
Alluvial diagram showing the flow of *E. coli* genomes from multilocus sequence type (ST, left) through O:H serotype (centre) to broad categories of antibiotic resistance mechanisms (right). Link thickness is proportional to the number of gene–isolate connections summarised for each ST–serotype–mechanism combination. The figure illustrates that a limited number of high-risk ST–serotype pairs (notably ST372/O83:H31 and ST372/O75:H31, as well as ST12 and ST131 carrying O4:H5 or O25:H4) account for most connections to efflux and regulatory mechanisms, while membrane/LPS changes, enzymatic modification and other mechanisms form smaller but lineage-specific streams, consistent with statistical tests that identify significant associations between several acquired AMR genes and particular STs and serotypes.

The alluvial plot (Figure 12) summarises the relationships between multilocus sequence type (ST), serotype and resistance mechanisms. The left side of the diagram shows that ST372, ST12 and ST131 account for the largest flows, with ST372 alone contributing numerous links to several serotypes, most prominently O83:H31 and O75:H31, but also O4:H31, O75:H5 and O8/O61:H19, consistent with its role as a highly versatile lineage capable of expressing multiple O:H combinations. ST12, ST131 and other extraintestinal pathogenic lineages (e.g., ST929, ST491, ST537, ST2015) also connect to a restricted panel of serotypes, including O4:H5, O25:H4, O2:H14, O54:H45 and O8:H16. In the central part of the alluvial, these serotypes segregate into four main categories (efflux, regulation, membrane/LPS modification and enzymatic modification). Quantitatively, efflux dominates the resistome, with 1329 links (63.6% of all ST–serotype–mechanism connections) leading to this category, followed by regulatory mechanisms (512 links, 24.5%), whereas membrane/LPS changes (104 links, 5.0%) and enzymatic modification (31 links, 1.5%) constitute smaller but non-negligible components, and miscellaneous mechanisms account for 4.5% of links. Serotypes O4:H5 alone contributes 237 efflux, 89 regulation, 20 membrane/LPS, 20 “other” and 2 enzymatic-modification links, while O83:H31 and O75:H31 each contribute 161 efflux and 63 regulation links, with additional flows to membrane/LPS, enzymatic and other mechanisms. The alluvial plot depicts a hierarchical organisation of the resistome in which (i) a conserved backbone of efflux and regulatory genes pervades all lineages; (ii) certain ST–serotype combinations, especially ST372/O83:H31, ST372/O75:H31 and ST12 or ST131 carrying O4:H5 or O25:H4, act as hubs that concentrate both intrinsic and acquired resistance mechanisms; and (iii) enzymatic modification and membrane/LPS alterations are layered on top of this backbone in a lineage- and serotype-dependent fashion, generating the observed diversity of AMR profiles.

#### 2.6.2. Virulome

**Figure 13:**
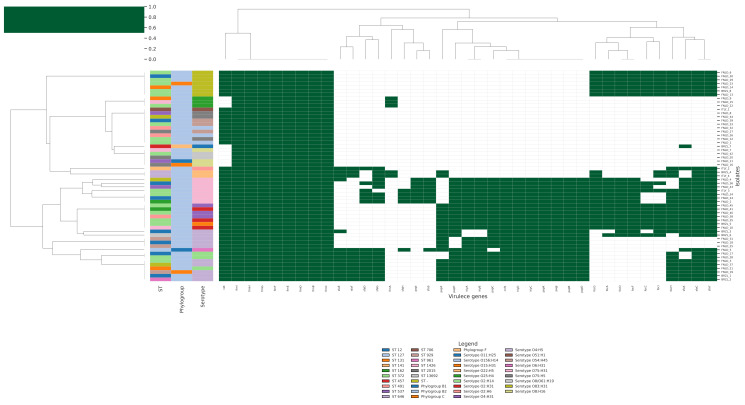
Heatmap showing presence (dark green) and absence (white) of selected virulence genes in genomes. Isolates are clustered according to virulence gene content and annotated by multilocus sequence type, phylogroup and serotype. A conserved core of type 1 fimbrial genes (*fimB–fimI*) is present in all genomes, whereas P, S and F1C fimbriae, haemolysin (*hlyABCD*), *cnf1* and *vat* form variably distributed modules that segregate with specific clades but show limited association with country of origin. Chi-square tests indicate that most virulence genes are homogeneously distributed among Italian, Brazilian and Finnish isolates, with significant enrichment observed only for a subset of colibactin (*clbJ*, *clbK*) and type II secretion genes (*gspC*, *gspD*, *gspF*, *gspH*), which likely reflect the uneven geographic distribution of particular high-risk lineages rather than strongly country-specific virulence profiles.

As supported by the available literature, adhesion and colonisation are the key event that drive the initial interface between *E. coli* and endometrial mucosa; for this reason, the virulome analyses are focused on adherence traits [14,23]. The virulence gene heatmap (Figure 13) reveals a highly structured and shared virulence repertoire among the 57 genomes, with a core block of adhesins and toxins present across phylogroups and serotypes and a second block of more variably distributed factors that stratify specific clades. The first block is dominated by the type 1 fimbrial operon (*fimB*–*fimI*), which is detected in all genomes, confirming its role as an essential colonisation factor in both intestinal and extra-intestinal settings. In contrast, the P fimbrial cluster (*papBCDHJK* and *papX*), S fimbriae (*sfaC/X/Y*, with lower frequencies for *sfaD–H/S*), F1C fimbriae (*focA–I*), and toxins such as the α-haemolysin operon (*hlyABCD*), cytotoxic necrotising factor 1 (*cnf1*) and vacuolating autotransporter toxin *vat* display intermediate prevalences (typically 40–80%), that correspond to canonical ExPEC virulence modules variably present in B2 and D phylogroups but are largely absent from commensal backgrounds. Notably, *vat* is present in 48/57 genomes (84.2%), underscoring its widespread contribution to pathogenic potential. When isolates are ordered according to hierarchical clustering, these variably distributed genes segregate into a few discrete virulence profiles that often mirror the underlying clonal structure (e.g., ST372, ST131, ST12), indicating that acquisition and loss of adhesin and toxin operons has occurred at the level of major lineages rather than individual genomes. The chi-square analysis for association with country of origin shows that most virulence genes do not differ significantly between Italian, Brazilian and Finnish isolates (*p* > 0.05 for the majority of loci, including *pap*, *sfa*, *foc*, *hly* and *vat*), suggesting that the observed lineage-level structuring is not strongly modulated by geography.

**Figure 14:**
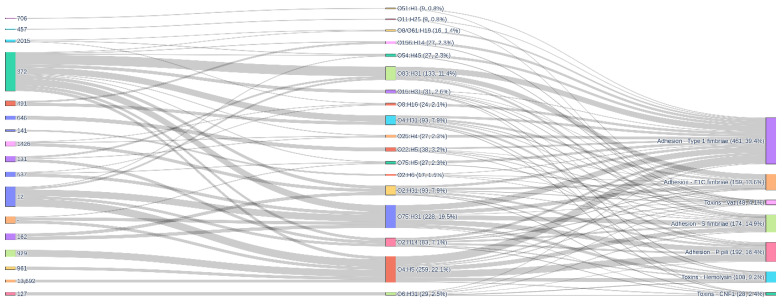
Alluvial diagram depicting flows from multilocus sequence type (ST, left) through O:H serotype (centre) to broad virulence mechanism categories (right) in the analysed genomes. Link thick-ness is proportional to the number of gene–isolate connections for each ST–serotype–mechanism combination.

The virulence alluvial (Figure 14) illustrates how a limited number of ST–serotype combinations concentrate the majority of adhesin and toxin functions in 57 *E. coli* strains. The left panel shows that ST372 and ST12 dominate the flow of isolates towards serotypes such as O83:H31, O75:H31 and O4:H5, with additional contributions from ExPEC-associated lineages including ST131, ST929 and ST491, reflecting the patterns observed in the core-genome phylogeny. In the central panel, these serotypes link into five mechanistic categories (type 1 fimbriae, P pili, S fimbriae, F1C fimbriae and toxins) where type 1 fimbrial adhesion clearly predominates, accounting for 461 links (39.4% of all ST–serotype–mechanism connections), followed by F1C fimbriae (159 links, 13.6%), P pili (192 links, 16.4%) and S fimbriae (174 links, 14.9%), while toxin categories are less frequent (Vat, 48 links, 4.1%; haemolysin, 108 links, 9.2%; CNF1, 28 links, 2.4%). O4:H5 is linked to 80 instances of type 1 fimbriae, 67 of P pili, 42 of S fimbriae and 14 of F1C fimbriae, together with 36 haemolysin, 10 CNF1 and 10 Vat-associated connections. On the other hand, O75:H31 and O83:H31 each contribute 56 type 1 and 49 F1C fimbrial links plus multiple toxin connections. Statistical tests confirm that many virulence genes are strongly associated with serotype (e.g., *fimA*, *cnf1*, *vat*, multiple *foc* and *pap* genes all have *p* ≤ 0.001 the gene–serotype relationship), while their association with ST is generally weaker and often non-significant, with only *fimA* and *sfaE* showing modest ST-level statistical significance (*p* ≈ 0.03 and 0.047), respectively.

**Figure 15:**
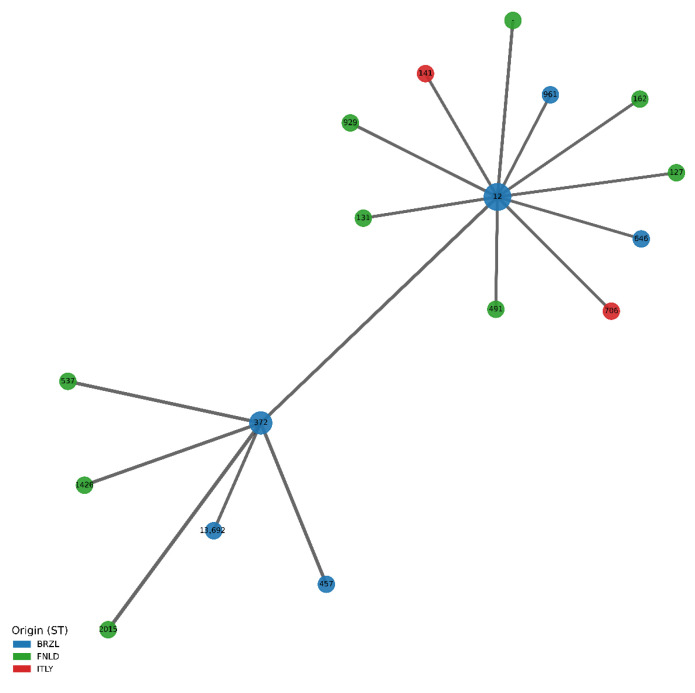
Minimum spanning tree (MST) of 17 multilocus sequence types (STs) constructed from pairwise distances based on shared gene content. Nodes are sized uniformly, coloured by sampling origin (BRZL, FNLD, ITLY or mixed origin), and annotated with the ST number; edges connect ST pairs included in the MST, with distances ranging from 5 to 17 units and 35–47 shared genes. ST12 (degree 11) and ST372 (degree 6) occupy central hub positions and are each detected in more than one country, linking multiple country-restricted leaf STs into a single network and indicating that a few widely distributed clonal lineages act as genomic backbones from which geographically biassed satellite lineages have emerged.

The minimum spanning tree (MST, Figure 15) provides a concise view of how the dominant clonal backgrounds are related by their shared gene content and how they connect isolates from different geographic origins.

The network comprises 17 ST nodes linked by 16 edges (average degree 1.88), with edge weights corresponding to pairwise distances derived from the number of shared genes; each edge label in the underlying table reports both the distance and the size of the common gene set. ST12 forms the main hub of the MST, with a degree of 11 and direct connections to 10 peripheral STs plus ST372, reflecting its role as a central, moderately diverged backbone lineage that retains 35–41 shared genes with its neighbours at distances of 11–17 units. The peripheral STs linked to ST12 (including ST131, ST929, ST491, ST646 and others) are almost all of Finnish origin, with a few Brazilian and Italian types (e.g., ST706, ST141) branching from the same node, indicating that multiple region-specific lineages radiate from a common genomic core rather than forming isolated country-specific clusters. ST372 constitutes a second major hub (degree 6) connected directly to ST12 and to five additional STs (ST2015, ST457, ST537, ST1426 and ST13692) via short edges corresponding to distances of 5–14 and up to 47 shared genes, consistent with ST372 acting as a highly connected, globally distributed ExPEC lineage. Notably, both ST12 and ST372 are annotated as mixed-origin nodes (BRZL; FNLD and BRZL; FNLD; ITLY, respectively), underscoring their role as transnational clonal backbones that bridge the Finnish, Brazilian and Italian subpopulations, whereas most other STs appear as leaf nodes restricted to a single country.

## 3. Discussion

Pyometra, a serious uterine infection that mostly affects intact female dogs and, to a lesser extent, female cats, is considered a medical emergency because it can quickly lead to sepsis, and death if not treated. Pyometra is treated surgically (ovariohysterectomy), but pre- and post-operative antimicrobial treatment is necessary to manage systemic infection and prevent peritonitis and septic shock. This study provides, to our knowledge, the first Italian whole-genome investigation of *Escherichia coli* isolated from canine and feline pyometra and contextualised within a broader international collection of pyometra-associated strains. The four Italian isolates (two isolated from dogs and two from cats) belong to phylogroup B2 and to sequence types ST706/O51:H1, ST141/O2:H6, ST372/O75:H31 and ST646/O22:H5, all lineages previously associated with extraintestinal pathogenic *E. coli* (ExPEC) in companion animals and, in the case of ST141 and ST372, also in humans. This mirrors recent WGS-based studies from Finland and Brazil, where B2-ExPEC dominate the *E. coli* population in canine pyometra and uterine infections and frequently share clonal backgrounds with uropathogenic *E. coli* (UPEC) from dogs and people [24]. By demonstrating that Italian pyometra cases are caused by the same high-risk B2 lineages that circulate internationally, our data extend this body of work geographically and emphasise that Italy participates in a wider One Health reservoir of ExPEC shared between dogs, cats and humans.

The antimicrobial susceptibility profile of the Italian isolates highlights an evolving but still exploitable therapeutic landscape. Phenotypic testing revealed uniform resistance to aminopenicillins and to several first- and third-generation cephalosporins, including cefovecin, together with complete resistance to nitrofurantoin, whereas no resistance was detected to fluoroquinolones, aminoglycosides, piperacillin, cefoperazone or trimethoprim–sulphonamide. This pattern is consistent with the growing European literature describing β-lactam-resistant and ESBL-producing *E. coli* in canine pyometra and UTIs, but contrasts with older studies in which aminopenicillins and early-generation cephalosporins remained predictably active [25,26,27]. At the genomic level, the Italian isolates share with Brazilian and Finnish genomes a highly conserved intrinsic resistome composed of multidrug efflux systems (*AcrAB-TolC*, *MdtEF-TolC*, *MdtABC-TolC*, *EmrAB-TolC*), global regulators (*marA*, *soxS*, *CRP*, *CpxA*) and lipid A modification pathways (*eptA*, *pmrF*, *ugd*), which together explain the broad baseline tolerance to multiple drug classes observed across the 57-genomes. In contrast, classical acquired ARGs (e.g., *CMY*-type and *TEM*-1 β-lactamases, plasmid-borne aminoglycoside phosphotransferases, *sul2* and *dfrA5*, and tetracycline efflux pumps) are sparse and unevenly distributed, reaching higher frequencies in specific Brazilian lineages but remaining sporadic in the Italian strains. The sraX pipeline further shows that putative resistance-associated SNPs are mostly located in chromosomal protein-coding and regulatory genes (e.g., *gyrA*, *parC*, *parE*, *ompF*, *folP*, fosfomycin transporter regulators) rather than in rRNA loci, suggesting a stepwise accumulation of mutations in intrinsic systems rather than widespread acquisition of horizontally transferred resistance cassettes [28,29,30]. Thus, the Italian pyometra isolates exemplify a scenario in which high-risk B2 lineages have already lost susceptibility to key β-lactam agents and nitrofurantoin but still lack many of the plasmid-encoded ESBLs and fluoroquinolone resistance determinants that dominate human ExPEC, particularly ST131 and ST1193. From a clinical standpoint, this supports continued use of fluoroquinolones, aminoglycosides and potentiated sulphonamides as effective rescue options for severe pyometra, while underscoring the need for stewardship to prevent the convergence of virulence and broad-spectrum resistance that is now characteristic of pandemic human ExPEC clones. The International Society for Companion Animal Infectious Diseases (ISCAID; https://www.iscaid.org/guidelines, accessed on 20 November 2025) and the British Small Animal Veterinary Association (BSAVA; https://www.bsava.com, accessed on 20 November 2025) guidelines suggest that empirical antimicrobial therapy for pyometra should be informed by local susceptibility patterns, with amoxicillin–clavulanic acid or a first-generation cephalosporin (e.g., cefalexin) frequently recommended as initial options due to their effectiveness against most *E. coli* strains and minimal risk of fostering resistance. The present findings endorse the ongoing use of aminopenicillins and potentiated aminopenicillins as empirical treatment for pyometra, with the progression to broader-spectrum drugs reserved for refractory cases or instances where susceptibility statistics reveal resistance.

Across the 57 genomes, the multilocus sequence types and O:H serotypes reveal a population structure dominated by well-known ExPEC B2 lineages, within which the four Italian pyometra isolates represent a geographically novel but genetically typical subset. The Italian strains belong to ST706/O51:H1, ST141/O2:H6, ST372/O75:H31 and ST646/O22:H5, all within phylogroup B2 and carrying canonical ExPEC virulence modules; among these, ST372/O75:H31 and ST141/O2:H6 are high-risk clones already reported from canine and human extraintestinal infections, while ST646/O22:H5 and ST706/O51:H1 extend the diversity of B2 backgrounds associated with pyometra [31,32,33]. The Brazilian and Finnish genomes are likewise enriched for B2 ExPEC STs (most prominently ST372, ST12, ST131 and related types) paired with serotypes such as O4:H5, O25:H4, O83:H31 and additional O75 and O2 variants that are well recognised in canine UPEC and human UPEC/ExPEC. Phylogenetic and MST analyses show that these ST–serotype combinations form mixed-origin clades and networks. ST372 and ST12 act as central hubs linking Brazilian, Finnish and Italian isolates, indicating that a limited number of internationally disseminated B2 lineages underpin pyometra and urinary infections across countries, while country-restricted STs and serotypes appear mainly as peripheral branches carrying region-specific accessory gene sets. In this context, the Italian ST/serotype repertoire does not represent a distinct local lineage, but rather documents that globally circulating ExPEC clones (especially ST372 and ST141 with their characteristic O75:H31 and O2:H6 serotypes) are now established in Italian companion animals and participate in the same transnational ExPEC gene pool as the Brazilian and Finnish strains [24,31,33].

Pan-genome analyses indicate that the genome collection has an open and structured pan-genome, composed of roughly 3000 strict-core genes shared by nearly all isolates and a very large accessory fraction partitioned into shell- and cloud gene families. When genomes are ordered by geographic origin, accessory genes form partially distinct patterns: Brazilian isolates carry the densest shell- and cloud repertoires, Finnish isolates are comparatively accessory-sparse, and the four Italian isolates are intermediate, sharing some Brazilian-associated modules but also harbouring their own low-frequency cloud families. These observations mirror reports from other regions in which ExPEC populations are structured by both clonal lineage and geography, with local accessory gene pools shaped by antimicrobial usage, host demography and circulation of mobile elements [24,31,34,35]. A minimum spanning tree of multilocus sequence types shows that ST372 and ST12 act as central hubs connecting multiple country-specific STs by short gene-content distances, supporting previous work that identified ST372 as the dominant canine ExPEC lineage globally and as a sporadic cause of human extraintestinal infection [31,36,37]. In this context, our finding of ST372/O75:H31 in an Italian dog with pyometra adds to accumulating evidence that this lineage represents a genuine One Health concern, with potential for bidirectional transmission between pets and humans.

The virulence gene repertoire of the Italian pyometra isolates is indistinguishable from that of high-risk ExPEC described elsewhere. Virulence profiling showed that all Italian genomes encode a full complement of adhesins (type 1 fimbriae, P, S and F1C pili), multiple iron acquisition systems (yersiniabactin, salmochelin, aerobactin, enterobactin and heme uptake), serum resistance factors, capsule and O-antigen biosynthesis genes, motility and chemotaxis loci, and toxins including colibactin, vat, haemolysin and CNF1. These traits correspond closely to those reported from canine pyometra and UTI isolates in Brazil, Scandinavia and Central Europe, where phylogroup B2 strains carrying pap, sfa/foc, hlyA, cnf1 and usp predominate in uterine contents and are significantly enriched in dogs with pyometra compared to healthy controls [38,39,40,41,42,43]. Similar virulence profiles, particularly in ST372 and ST141, have been described among canine and human UPEC, reinforcing their zoonotic potential.

## 4. Materials and Methods

### 4.1. Culture Conditions and Bacterial Identification

Samples were obtained at “Clinica Veterinaria Citta’ di Saronno” during 2023–2024, after elective ovariohysterectomy in two dogs and two cats due to the diagnose of pyometra. The surgeries were performed in a sterile surgical room; the patients had received an antibiotic treatment before the surgery. After the surgery, the uterine horns were incised with scalpel blade and the content of the organ was sampled utilising transport swabs containing Amies medium. Upon collection, the samples were promptly refrigerated and dispatched to the Laboratory of Microbiology, Genomics, and Proteomics of Microorganisms of Human, Animal, Food, and Environmental Origin within the Department of Biomedical, Surgical, and Dental Sciences at the University of Milan, where they were isolated and subsequently identified.

Upon arrival, samples were inoculated onto Tryptic Soy Agar (TSA; Microbiol, Uta, Italy) supplemented with 5% defibrinated sheep blood (ThermoFisher, Monza, Italy) and incubated aerobically at 37 °C for 24 h. Subsequently, colonies that exhibited characteristics such as appearance, odour, morphology, and haemolysis indicative of Enterobacteriaceae were inoculated onto MacConkey Agar (Difco, CO, USA) and incubated aerobically at 37 °C for 24 h. Following incubation, isolated colonies were transferred to 10 mL of Brain-Heart infusion broth (BHI, Microbiol, Uta, Italy) and subjected to further incubation under same conditions as previously described. Each sample was cryopreserved at −20 °C in triplicate within a BHI broth solution containing 25% (*v*/*v*) glycerol. Before use, 50 µL of each stock culture was inoculated onto TSA (Microbiol, Uta, Italy) supplemented with 5% defibrinated mutton blood (ThermoFisher, Italy) and incubated aerobically at 37 °C for 24 h.

### 4.2. Antimicrobial Testing

After identification, the determination of the antimicrobial resistance profile was achieved with the Kirby–Bauer disc diffusion assay following the Clinical and Laboratory Standard Institute guidelines. Briefly, 3–4 isolated colonies of each strain were resuspended in sterile saline solution (NaCl 0.8%) to reach 0.5 McFarland Unit (equivalent to 1.5 × 10^8^ Colony-Forming-Unit/mL) and immediately dispersed on a Mueller-Hinton Agar (Microbiol, Uta, Italy) plate. The following antibiotic discs were applied on the surface of the plate using sterile forceps: amoxicillin 10 µg, amoxicillin/clavulanic acid 30 µg, ampicillin 10 µg, cefovecin 30 µg, cefadroxil 30 µg, ceftriaxone 30 µg, cephalexin 30 µg, cephazolin 30 µg, amoxicillin/clavulanic acid 30 µg, marbofloxacin 5 µg, pradofloxacin 5 µg, enrofloxacin 5 µg, gentamicin 10 µg, ciprofloxacin 5 µg, piperacillin 30 µg, kanamycin 30 µg, trimethoprim/sulphamethoxazole 25 µg (19:1), moxifloxacin 5 µg, levofloxacin 5 µg, cefoperazone 30 µg, and nitrofurantoin 100 µg. The plates were incubated aerobically at 37 °C for 24 h and then inhibition halos were measured.

### 4.3. DNA Extraction, Nanopore Sequencing and Bioinformatic Analysis

The MinION Mk1C platform (Oxford Nanopores Technologies, Oxford, UK) was employed to sequence all *E. coli* strains using a long-read sequencing (LRS) approach. The Quick-DNA^TM^ HMW MagBead Kit (Zymo Research, Irvine, CA, USA) was used to extract high-molecular-weight DNA from a fresh culture of *E. coli* on blood agar. The colonies were resuspended in 500 µL of PBS (Phosphate-Buffered Saline, Euroclone, Milan, Italy) and centrifuged at 10,000× *g* for 1 min at room temperature. The NanoReady Touch series Micro Volume (UV-Vis) (Aurogene, Roma, Italy) was employed to evaluate the DNA quantity and quality, assuring that the A_260_/A_280_ and A_260_/A_230_ ratios were within the range of 1.8 and 2, respectively.

The bioinformatics pipelines, parameters for script configuration, and analyses conducted for bacterial assembly identical to those in the prior publication [44]. For comparative genomics and pan-genome analyses, we combined several open-source pipelines, each run with their current stable release from the respective GitHub repositories. The pan-genome was first inferred with PIRATE v1.0.4 (https://github.com/SionBayliss/PIRATE), which was used to cluster homologous coding sequences into gene families across all genomes and to estimate allelic diversity and duplication/fission events. Core-genome alignment and refinement of gene clusters were performed with Panaroo v1.3.3 (https://github.com/gtonkinhill/panaroo) and PGAP2 (https://github.com/bucongfan/PGAP2), whose post-processing modules were applied to partition gene families into strict-core, soft-core, shell- and cloud components and to generate rarefaction curves. A complementary partitioned pan-genome graph and prediction of regions of genomic plasticity were obtained with PPanGGOLiN v1.2.1 (https://github.com/labgem/PPanGGOLiN). For interactive visualisation of gene-presence/absence matrices, average nucleotide identity and functional annotation, we used Anvi’o v8 (https://github.com/meren/anvio). Whole-genome similarity based on k-mer sketches was estimated with sourmash v4.8.6 (https://github.com/sourmash-bio/sourmash), which provided an additional distance matrix for clustering. Antimicrobial resistance genes were screened with sraX v1.5.3 (https://github.com/lgpdevtools/sraX) and ResFinder v4.3.1 (https://github.com/cadms/resfinder), while additional acquired ARG and virulence loci were detected with Abricate v1.0.1 (https://github.com/tseemann/abricate) against the CARD and VFDB databases. Secondary metabolite biosynthetic gene clusters were predicted using antiSMASH v6.0 (https://github.com/antismash/antismash). For MLST, phylogroup and serotype assignment of *E. coli*, we used ClermonTyping (https://github.com/A-BN/ClermonTyping) and ECTyper (https://github.com/digitalcytometry/ecotyper), whereas functional classification of protein-coding genes into COG categories was carried out with COGclassifier (https://github.com/aleimba/bac-genomics-scripts). Gene-by-gene and core-genome phylogenies and additional clustering (e.g., Roary, panaroo2, PanViTa, IPGA) were generated with the corresponding tools installed from their official GitHub repositories, and all software was executed under a Linux environment using conda/mamba-managed environments to ensure full reproducibility.

### 4.4. Statistical Analysis

Statistical analyses were performed in Python 3.11 using pandas, SciPy and seaborn/matplotlib. Presence–absence matrices for AMR and virulence genes and binary phenotypic resistance data were derived from the metadata table, and resistance frequencies were calculated as proportions of resistant isolates for each antibiotic. Gene–country associations (Brazil, Finland, Italy) were evaluated by constructing contingency tables of gene presence versus country and applying Pearson’s chi-squared test (scipy.stats.chi2_contingency); for each gene we recorded χ^2^ statistic, degrees of freedom, *p*-value, number of isolates and minimum expected cell count, flagging tests with any expected cell < 5 as exploratory. Additional associations between individual genes and multilocus sequence type or serotype were tested using chi-squared or Fisher’s exact tests, as appropriate, and *p*-values were exported for descriptive interpretation without formal correction for multiple testing. Hierarchical clustering of gene presence–absence matrices (average linkage, Euclidean distance) was used to generate annotated heatmaps, while aggregated counts of genes per functional category (e.g., efflux, regulation, membrane/LPS modification, toxins, adhesins) were used to build alluvial/Sankey plots linking sequence type, serotype and mechanism, with link thickness proportional to the number of gene–isolate connections.

## 5. Conclusions

By integrating these genomic findings with detailed phenotypic data and by com-paring Italian isolates to well-characterised Brazilian and Finnish collections, this work extends previous regional studies and fills an important gap in Italian veterinary microbiology, where WGS has so far been applied mainly to ExPEC from healthy animals and food rather than to clinical pyometra isolates. The main limitations include the small number of Italian genomes and the focus on a single clinical syndrome; nonetheless, the concordance between our observations and larger international datasets suggests that the trends we document (B2 dominance, high-risk STs shared with UTI, an intrinsic resistome, and a large, open accessory genome) are robust features of pyometra-associated *E. coli*. Future work should build on this foundation by expanding sampling within Italy, integrating longitudinal data on antimicrobial use, and explicitly testing for household-level sharing of ST372, ST141 and related lineages between dogs, cats and owners, as recently demonstrated in other European settings. In the meantime, our results underscore the need for updated, genomics-informed empirical treatment guidelines for pyometra, for systematic surveillance of ExPEC in companion animals, and for closer coordination between veterinary and human health sectors to monitor and mitigate the zoonotic risks posed by these increasingly complex *E. coli* populations.

## Figures and Tables

**Table 1:** Genetic characteristics of sequenced strains.

Name	Source	Age	Y. of Isol.	GenBank	Assembly	Comp (%)	Cont (%)	Cov	Serotype	Phylogroup	MLST	Genome Size (Mbp)	GC %	N. Contigs	CDS	tRNA	rRNA
ECOPI1	Cat	na	28-09-2023	GCA_049311655.1	ASM4931165v1	100.0	0.49	172	O51:H1	B2	706	4.95	51	4	4545	89	22
ECOPI2	Cat	18 months	05-04-2024	GCA_049544955.1	ASM4954495v1	100.0	0.95	139	O2:H6	B2	141	5.13	51	1	4712	87	22
ECOPI3	Dog	13 years	29-05-2024	GCA_049544985.1	ASM4954498v1	100.0	1.37	135	O75:H31	B2	372	5.15	50	1	4755	86	22
ECOPI4	Dog	6 years	15-10-2024	GCA_049544965.1	ASM4954496v1	100.0	0.85	86	O22:H5	B2	646	5.25	50	1	4827	88	22

Abbreviations: Y. of isol. = year of isolation; Comp = completeness; Cont = contamination; Cov = coverage; CDS = coding sequences; na = not available.

## Data Availability

All the sequences were deposited under the BioProject PRJNA1243058 (https://www.ncbi.nlm.nih.gov/bioproject/PRJNA1243058).

## Supplementary Materials

The following supporting information can be downloaded at https://www.mdpi.com/article/10.3390/antibiotics15020212/s1, Figure S1: Stacked bar plots summarising sraX results for *E. coli* genomes; Table S1: genomic characteristics of Finnish and Brazilian strains.
