# Supplementary material for: Genomic Characterisation of Pyometra-Associated Escherichia coli in a Lombardy Veterinary Clinic: A Nanopore-Based Case Series
Source: Antibiotics (Basel). 2026 Feb 15;15(2):212. doi: 10.3390/antibiotics15020212 (PMC12937276; doi:10.3390/antibiotics15020212)
Supplement: Supplementary file 1 [file antibiotics-15-00212-s001.zip › Supplemental Figure S1.pdf]

Supplemental Figure S1. Stacked bar plots summarising sraX results for *E. coli* genomes. (A) Proportion of antibiotic resistance gene (ARG) classes per genome based on unique ARGs, showing a conserved efflux- and AmpC-dominated backbone with low-frequency acquisition of additional β-lactam, aminoglycoside, sulfonamide, trimethoprim and tetracycline determinants. (B) Type and fraction of ARGs carrying putative resistance-associated SNPs per genome, indicating that relatively few ARGs are mutated and that most variants map to chromosomal protein-coding or regulatory genes rather than rRNA loci or overexpression-linked determinants.

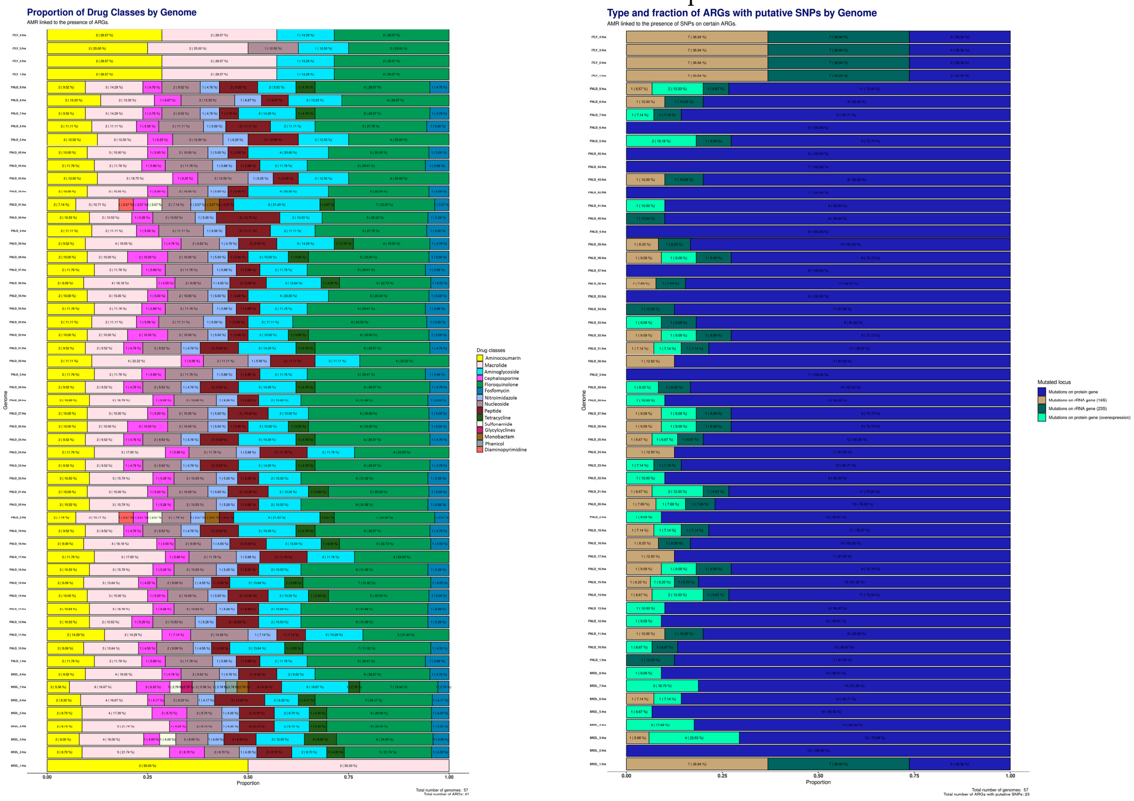

A

B
